# IgG to Galactose-Alpha-1,3-Galactose: Impact of Alpha-Gal IgE Sensitization, Blood Type, and Tick Bites

**DOI:** 10.3390/antib14020043

**Published:** 2025-05-16

**Authors:** Samuel M. Ailsworth, Matthew MacCallum, Nathan E. Richards, Lisa J. Workman, Pamela Schoppee Bortz, Thomas Makin, Thomas A. E. Platts-Mills, Jeffrey M. Wilson

**Affiliations:** Division of Allergy and Clinical Immunology, Department of Medicine, University of Virginia, Charlottesville, VA 22908-1355, USAmhm7mm@virginia.edu (M.M.);

**Keywords:** alpha-gal, IgE, IgG, red meat allergy, ticks

## Abstract

**Background:** Antibodies to galactose-alpha-1,3-galactose (alpha-gal), particularly the IgM and IgG isotypes, are abundant in human sera. These antibodies are known to be an important xenotransplantation barrier, but the full implications of these antibodies to health and disease remain incompletely understood. By contrast, IgE to alpha-gal is uncommon in the population but has been associated with tick bites and causally linked with mammalian meat allergy, often now known as alpha-gal syndrome (AGS). To date, there have been few population-based studies that have investigated alpha-gal IgG levels in relation to demographic factors, diet, tick bites, and mammalian meat allergy. **Methods:** Adults, predominantly healthcare workers, were recruited for a COVID-19 vaccine study. At least one serum sample was collected, and subjects completed questionnaires to provide demographic, diet, and tick exposure data. Alpha-gal IgG, IgE, and total IgG were measured using the ImmunoCAP platform, and blood group was assessed via reverse typing using stored serum. We also assessed alpha-gal IgG levels among subjects with AGS, recruited from an allergy clinic. **Results:** The median age of the 267 subjects in the vaccine cohort was 42 years, and median alpha-gal IgG levels were 3.0 μg/mL. Alpha-gal IgG levels were higher among the 43 (16.1%) subjects who had alpha-gal IgE sensitization (≥0.1 IU/mL) and among subjects lacking the B blood group antigen (blood groups A and O). Alpha-gal IgG levels did not differ between the subjects who had asymptomatic alpha-gal IgE sensitization and those who had meat allergy. However, both groups had higher alpha-gal IgG levels than subjects who lacked alpha-gal IgE sensitization. Subjects who reported prior tick or chigger bites had higher alpha-gal IgG levels than those without a bite history, regardless of alpha-gal IgE sensitization status. **Conclusions:** In a population-based cohort, alpha-gal IgG antibodies were found to be prevalent, and levels were increased in subjects with blood groups A and O, subjects who were alpha-gal IgE sensitized, and those who reported a history of tick bites.

## 1. Introduction

Galactose-alpha-1,3-galactose (alpha-gal) is an oligosaccharide, expressed by non-primate mammals and new world monkeys, of increasing biomedical interest. First described almost a century ago as a “B-like” (blood group) antigen, it has long been apparent that humans, who universally lack the enzymatic machinery to synthesize alpha-gal, produce abundant anti-alpha-gal IgG, as well as IgM and IgA antibodies [1,2,3]. Some estimates suggest that anti-alpha-gal IgG could represent up to, or exceed, 1% of the total IgG repertoire in adults, though other estimates are lower [4,5]. The induction of these anti-alpha-gal antibodies is thought to result from regular exposure to alpha-gal present on the commensal gut microbiome [6,7].

The relevance of these antibodies to health and disease is incompletely understood. IgM and IgG antibodies to alpha-gal are known to be an important barrier to xenotransplantation of solid organs from pigs and other non-primate mammals into humans [8,9]. There are also theoretical risks that anti-alpha-gal antibodies could contribute to the premature failure of biologic valves and grafts that are mammalian-derived [10,11,12]. More recently, IgE antibodies to alpha-gal were discovered to be an important cause of allergic reactions to mammalian-sourced food and products [13,14]. Often referred to as alpha-gal syndrome (AGS), symptoms such as urticaria, angioedema, and gastrointestinal (GI) symptoms are classically delayed 3–5 h following oral consumption of mammalian meat [15,16]. Connections between alpha-gal IgE and cardiovascular disease have also been reported [17,18]. Alpha-gal IgE sensitization is only observed in select individuals, and there is now convincing evidence that bites from certain ticks, including *Amblyomma americanum* (lone star tick) in the United States, are an important cause of alpha-gal IgE induction, though other parasites may also contribute to IgE sensitization [19,20,21,22,23,24,25]. Further solidifying a link between tick exposure and alpha-gal, a recent report indicates that tick bites can promote alpha-gal IgG, even in individuals who do not make alpha-gal IgE [26].

To date, there have been few population-based studies that have quantified alpha-gal IgG levels in relation to demographic traits and other potential risk modifiers such as ABO blood group and diet. Here, we took advantage of a cohort of 267 adults recruited as part of a COVID-19 vaccine study and used a quantitative ImmunoCAP-based assay to address these questions. The alpha-gal IgG assay was validated using two different alpha-gal glycoproteins on the ImmunoCAP solid phase. The results from this “random” adult cohort were compared with a group of AGS patients recruited from the allergy clinic who were positive for alpha-gal IgE and had convincing histories of mammalian meat allergy (MMA).

## 2. Materials and Methods

### 2.1. COVID-19 Vaccine Cohort

Subjects from the University of Virginia (UVA) and the Charlottesville, Virginia area were recruited to take part in a COVID-19 vaccine study between 2020 and 2022, as previously reported [27,28]. This cohort was approved by the UVA Institutional Review Board, and all participants provided written and verbal consent. The majority of the cohort was comprised of healthcare workers from the University of Virginia hospital, though eligibility expanded to members of the Charlottesville community in fall 2021. Subjects were recruited because of their interest in learning about their COVID IgG antibody levels following vaccination. Therefore, there was no selection for subjects on the basis of allergic disease or MMA. Subjects were recruited by email and flyer to participate in a study in which they could find out their SARS-CoV-2 IgG antibody levels following primary series and booster vaccines. Blood and questionnaire data were obtained at each visit. Subjects who provided at least one blood sample and completed questionnaires were included in this analysis. A subset of 207 subjects completed a detailed questionnaire that elicited data such as diet, tick exposure, and GI symptoms. Blood samples were processed, and sera were stored at −30 °C until being assayed. The same serum sample was used for IgE and IgG assays, while blood typing and total IgG measurements were conducted on the serum samples with the most volume available.

### 2.2. AGS Patient Cohort

Patients with a convincing history of MMA and positive alpha-gal IgE test were recruited from the University of Virginia Allergy clinic as part of a UVA Institutional Review Board-approved study. A detailed questionnaire elicited data, including information on diet and allergic symptoms. Serum samples were biobanked at −30 °C prior to antibody assays.

### 2.3. Alpha-Gal IgE, Total IgE, and ABO Assays

Alpha-gal specific IgE (sIgE) antibodies and total IgE antibodies were measured via ImmunoCAP 250 (Thermo Fisher/Phadia, Waltham, MA, USA) using commercially available reagents, and results were expressed in IU/mL. Alpha-gal sIgE levels were assayed using the bovine thyroglobulin ImmunoCAP test (o215). Specific IgE levels were considered positive if ≥0.1 IU/mL, though measurements as low as 0.01 IU/mL were used to calculate sIgG/sIgE ratios. For calculation of sIgG/sIgE ratios, sIgE levels were converted to ng/mL using the conversion 1 IU/mL = 2.4 ng/mL [29]. ABO blood group status was determined by reverse-typing using commercially available reference cells (Bio-Rad, Dreieich, Germany) according to the manufacturer’s instructions.

### 2.4. IgG Assays

Alpha-gal-specific IgG (sIgG) antibodies were measured using ImmunoCAP 250. Alpha-gal linked to human serum albumin (HSA) (Dextra, Reading, UK) was biotinylated, and 2 μg was bound to a streptavidin ImmunoCAP, as previously described [30]. Each sample was run in parallel with an unconjugated streptavidin ImmunoCAP, and results from this control test were subtracted from the alpha-gal-HSA ImmunoCAP to account for background binding. Any sample where the alpha-gal HSA result was <0.05 μg/mL greater than the streptavidin control was considered under the assay detection limit and assigned a value of 0.05 μg/mL for analysis. For a subset of samples, alpha-gal IgG was also measured with the commercially available bovine thyroglobulin (o215) ImmunoCAP test. Of note, this o215 test uses a chemistry that does not rely on streptavidin-biotin binding. Specific IgG levels were expressed in μg/mL and were converted to ng/mL for sIgG/sIgE ratio calculations. Total IgG was measured in 93 subjects using ImmunoCAP, as previously reported [31].

### 2.5. Statistical Analysis

Antibody levels were expressed as geometric mean with 95% confidence interval. Categorical variables were compared using Pearson’s Chi-Square test. Correlation was assessed among log-transformed antibody levels using Spearman’s correlation coefficient. Mann–Whitney U test and Kruskal–Wallis test were used to compare continuous data between groups. Multiple linear regression models were used to compare associations between demographic variables and log-transformed antibody levels. Optimal antibody thresholds for predicting symptomatic MMA were determined using receiver operator characteristic (ROC) analysis and Youden Index. Statistical analysis was performed using GraphPad Prism 9 (GraphPad Software, San Diego, CA, USA).

## 3. Results

### 3.1. Demographics and Alpha-Gal Antibody Responses in the Vaccine and AGS Patient Cohorts

A total of 267 subjects were recruited as part of a COVID-19 vaccine study in the Charlottesville, Virginia area. Median age was 42 (IQR 31–55) years, 202 subjects (75.7%) were female, and the majority of subjects (80.9%) reported White race (Table 1). As previously reported, using a cutoff of 0.1 IU/mL, IgE sensitization to alpha-gal was detected in 43 subjects (16.1%) [28]. Of those who were sensitized, the alpha-gal sIgE geometric mean (GM) level was 0.76 IU/mL (95% CI 0.44–1.28), and seven reported symptomatic MMA. Alpha-gal IgG levels using the alpha-gal HSA assay ranged from beneath the detection limit of the assay (*n* = 22) to 53.4 μg/mL, with a median level of 3.0 μg/mL (IQR 1.2–5.8). Total IgG was measured in a convenience sample of 93 subjects. The median total IgG level among these was 1010 mg/dL (IQR 859–1170 mg/dL). The corresponding median alpha-gal specific IgG as a percentage of total IgG was 0.03% (IQR 0.009–0.06%).

The AGS patient cohort included 38 patients recruited from the allergy clinic for symptoms and lab testing consistent with AGS. The median age was 62 (IQR 52–73) years, 18 subjects (47.4%) were female, and the majority of subjects (76.3%) reported White race. In this cohort, alpha-gal IgG levels with the alpha-gal HSA assay ranged from 0.93 μg/mL to 20.6 μg/mL, with a median level of 9.2 μg/mL (IQR 5.4–15.7).

### 3.2. Alpha-Gal ImmunoCAP Assays Using Alpha-Gal HSA and BTG Yield Similar Results

**Figure 1:**
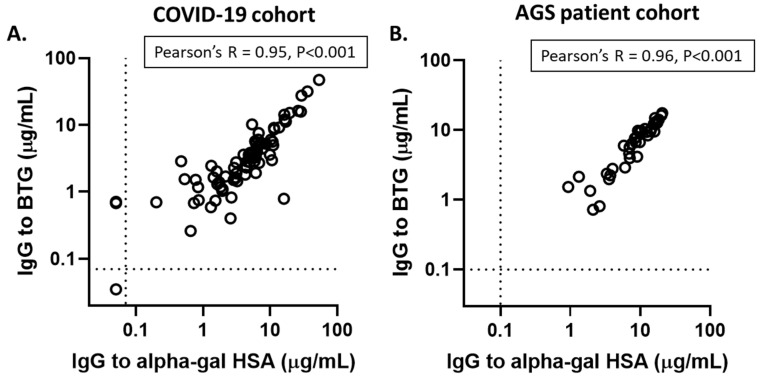
Comparison of alpha-gal IgG assay results (μg/mL) using alpha-gal HSA on the solid phase and results from the commercially available Thermo-Fisher bovine thyroglobulin alpha-gal assay (o215). (**A**) Alpha-gal IgG levels measured among 81 samples from the COVID-19 vaccine cohort. (**B**) Alpha-gal IgG levels measured among samples from 38 AGS patients.

To provide additional validation for the alpha-gal IgG assay (which used alpha-gal HSA as the solid-phase), a convenience sample was also tested with the commercially available alpha-gal ImmunoCAP, which uses beef thyroglobulin on the solid phase. Comparing measurements in 81 samples, of which 41 were alpha-gal IgE-positive, there was a very strong correlation between the two alpha-gal IgG assays (R = 0.95, *p* < 0.001) (Figure 1A). A strong correlation between the two assays was also observed in serum for the 38 AGS patients (Figure 1B).

### 3.3. Alpha-Gal IgG Levels in Relation to Age, Sex, Race, Diet, ABO Blood Group, and Alpha-Gal IgE Sensitization

**Figure 2:**
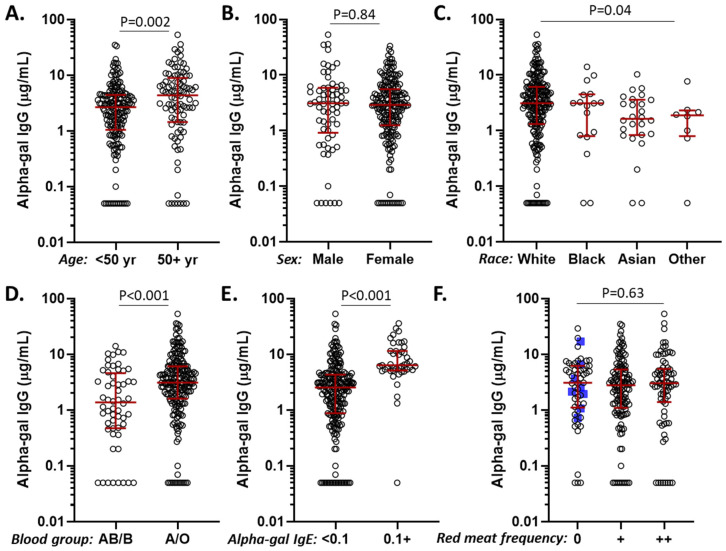
Alpha-gal IgG levels (μg/mL) among subjects in the COVID-19 vaccine cohort. (**A**) Alpha-gal IgG levels stratified by age. (**B**) Alpha-gal IgG levels stratified by sex. (**C**) Alpha-gal IgG stratified by race. (**D**) Alpha-gal IgG levels stratified by blood group among those expressing the B antigen (AB/B) and those not expressing the B antigen (A/O). (**E**) Alpha-gal IgG levels stratified by alpha-gal IgE sensitization status. (**F**) Alpha-gal IgG levels stratified by mammalian meat consumption (“+” = 1–2 servings/week; “++” = ≥3 servings/week), with blue squares indicating those also avoiding dairy (*n* = 263 with complete dietary data set).

**Figure 3:**
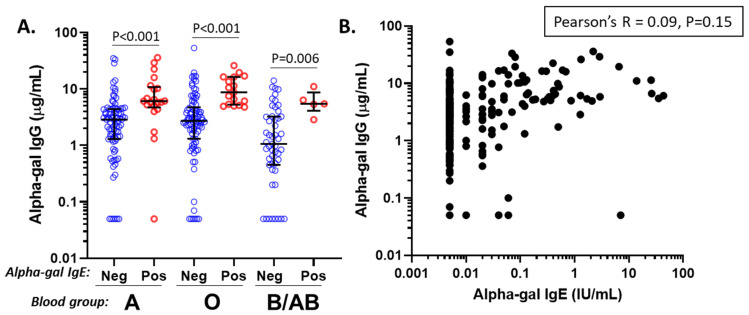
Alpha-gal IgG levels, (**A**) stratified by blood group status and alpha-gal IgE sensitization status, and (**B**) in relation to alpha-gal IgE levels (bottom left: *n* = 16 with undetectable alpha-gal IgG and alpha-gal IgE).

In the vaccine cohort, exploration of factors that could influence alpha-gal IgG levels revealed differences for age, race, ABO blood group, and alpha-gal IgE status, but not sex or frequency of mammalian meat consumption (Figure 2). Regression analysis including these variables confirmed that alpha-gal IgG levels were significantly higher among those who were alpha-gal IgE-sensitized (*p* < 0.001) and who were not B/AB blood group-positive (*p* = 0.002), but effects relating to age and race were no longer significant in the multiple variable model (Table 2). Stratification by ABO blood group and alpha-gal IgE sensitization demonstrated that B/AB subjects generally had low alpha-gal IgG levels, but sensitized B/AB subjects nonetheless had relatively high IgG levels (Figure 3A). Although the presence of IgE sensitization was strongly associated with high levels of alpha-gal IgG, there was no strong direct relationship between the levels of the two variables (Figure 3B).

### 3.4. Alpha-Gal IgG in Relation to Mammalian Meat Allergy

**Figure 4:**
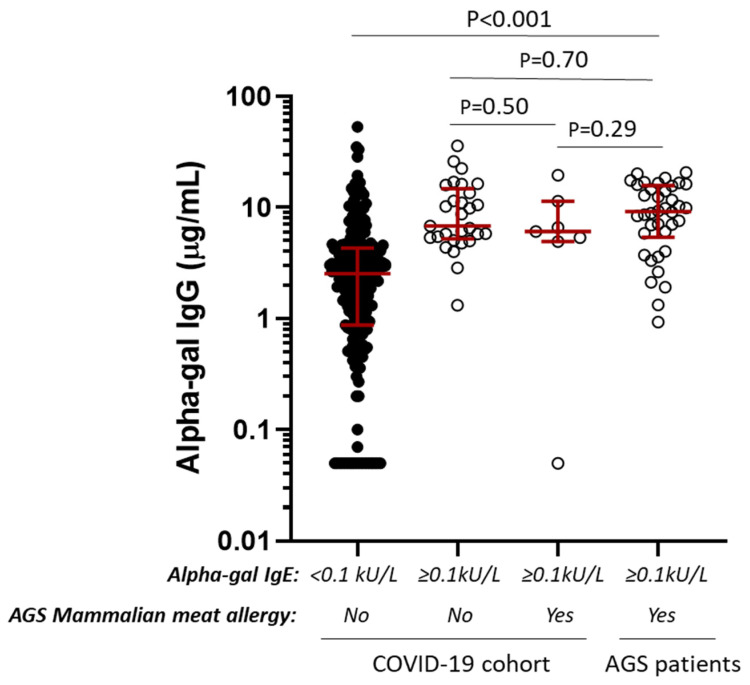
Alpha-gal IgG levels in relation to alpha-gal IgE sensitization and symptomatic mammalian meat allergy among the COVID-19 vaccine cohort and the AGS patient cohort.

Not all subjects who are alpha-gal IgE positive react to mammalian meat. In fact, some reports suggest that, in community or high-risk cohorts, upwards of 80–90% of those who are alpha-gal IgE sensitized do not have clinically relevant MMA [28,32]. Here, we sought to address whether alpha-gal IgG levels could discriminate between sensitized individuals with and without symptomatic MMA. Alpha-gal IgG levels were similar between 36 alpha-gal IgE-positive subjects who did not report MMA and 7 individuals with MMA (Figure 4). To further explore this, we measured alpha-gal IgG in 38 patients with symptomatic MMA recruited from the allergy clinic. The demographic features of this group, all of whom had detectable alpha-gal IgE, are shown in Table 1. Alpha-gal IgG levels in these 38 patients were similar to the levels in the sensitized subjects from the vaccine cohort, including 29 who did not have a history of MMA but reported routine consumption of mammalian meat.

### 3.5. Diagnostic Utility of Alpha-Gal IgE and IgG Antibody Levels

**Figure 5:**
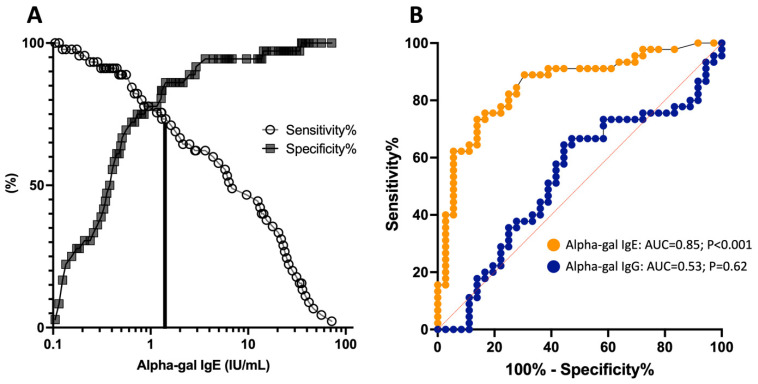
Receiver operating characteristic (ROC) analysis predicting symptomatic AGS among subjects with positive alpha-gal IgE results (IgE ≥ 0.1 IU/mL). (**A**) Optimal alpha-gal IgE cutoff and (**B**) ROC curves comparing the diagnostic value of alpha-gal IgE and IgG in predicting AGS.

In a prior analysis of the vaccine cohort, receiver operating characteristic (ROC) curves were used to determine an optimal alpha-gal IgE cutoff for predicting symptomatic MMA among sensitized subjects [28]. Here, we repeated this analysis with sensitized subjects from both the vaccine cohort and the AGS patient cohort, which provided a significantly larger number of symptomatic subjects (*n* = 45), compared to the prior analysis (*n* = 7). We then compared the performance characteristics of alpha-gal IgE and alpha-gal IgG for predicting symptomatic MMA among the alpha-gal sensitized individuals. ROC analysis revealed an optimal alpha-gal IgE cutoff of 1.4 IU/mL or greater, which was nearly identical to our previously determined cutoff of ≥1.5 IU/mL, using only the vaccine cohort. The area under the ROC curve (AUC) was 0.85 (95% CI 0.77–0.94, *p* < 0.001) for alpha-gal IgE levels. In contrast, alpha-gal IgG performance characteristics for predicting MMA (among those who were alpha-gal IgE positive) were inferior and not significant, with an AUC of 0.53 (95% CI 0.40–0.66, *p* = 0.62) (Figure 5). Of note, calculated alpha-gal sIgG/sIgE ratios were not more accurate at predicting MMA than alpha-gal IgE alone (AUC = 0.84 [95% CI 0.75–0.94], *p* < 0.001).

### 3.6. Tick Bites and Their Relation to Alpha-Gal IgE Sensitization and Alpha-Gal IgG Levels

**Figure 6:**
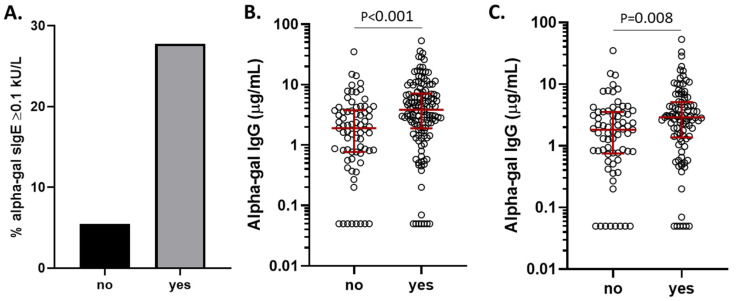
Alpha-gal IgE prevalence and IgG levels in relation to tick/chigger exposure. (**A**) The prevalence of alpha-gal IgE among 207 subjects in the vaccine cohort, based on their answer to questions regarding tick/chigger bite history. (**B**) The levels of alpha-gal IgG among 207 subjects in the vaccine cohort, based on their answer to questions regarding tick/chigger bite history. (**C**) Alpha-gal IgG levels among 166 subjects from the vaccine cohort who were not sensitized to alpha-gal (alpha-gal IgE < 0.1 IU/mL), stratified by tick/chigger bite history.

To explore the effects of tick bites on alpha-gal IgE and IgG antibodies, we analyzed data from the 207 subjects from the vaccine cohort who had completed a detailed questionnaire that asked about tick exposure. Among subjects who reported a history of “any tick or chigger bite” in the past, 37/134 (27.6%) had positive alpha-gal IgE levels, compared to 4/73 (5.5%) of subjects who denied any history of bites (*p* < 0.001, Figure 6A). Although technically “chigger” refers to the larval form of a trombiculid mite, we included it in our questionnaire as many patients who reside in Virginia and experience bites from larval stage ticks report them as chiggers [33]. Given the linked relationships between tick bites, alpha-gal IgE, and alpha-gal IgG, it was not surprising that alpha-gal IgG levels were higher among subjects reporting a tick bite history (Figure 6B). To investigate whether tick bites could have an effect on alpha-gal IgG that is independent of alpha-gal IgE sensitization, we narrowed our analysis to non-sensitized subjects who had completed the detailed questionnaire (*n* = 166). Even among subjects lacking alpha-gal IgE sensitization, subjects who reported a history of tick or chigger bites had significantly higher alpha-gal IgG levels (GM 2.28 μg/mL [95% CI 1.70–3.07]) than subjects who reported no history of bites (GM 1.3 μg/mL [95% CI 0.90–1.88]) (Figure 6B). We next assessed this finding using a multiple linear regression model to control for demographic factors and blood group. In this analysis, we found that a history of tick or chigger bites remained significantly associated with increased alpha-gal IgG levels when controlling for B blood group expression, age, sex, and race (Table 3).

## 4. Discussion

Despite the fact that IgG antibodies to alpha-gal were first reported a century ago, many open questions about their levels in blood and their relation with health and disease remain. Here, we employed a quantitative assay with standardized units and a well-characterized adult cohort to identify factors that promote alpha-gal IgG. The ImmunoCAP-based assay we used in this investigation to measure alpha-gal IgG followed established methods, including methods we developed and used to quantify anti-SARS-CoV-2 IgG among subjects in this same COVID-19 vaccine cohort [27,30]. The strong correlation between the alpha-gal IgG levels measured with two distinct sources of alpha-gal on the solid phase—alpha-gal HSA and beef thyroglobulin (o215)—speaks to the specificity and quantitative aspects of the assay for measuring antibodies to alpha-gal. We would also like to highlight that the two different assays used different chemistries to link the antigens to the solid phase.

We found that median alpha-gal IgG levels were 3.0 μg/mL (IQR 1.2–5.8) among members of the vaccine cohort, with the majority (92%) being in a detectable range. This is lower than some of the early estimates from Galili and colleagues, where alpha-gal IgG levels approximated 50 to 100 μg/mL, but fairly comparable with a report by Yu and colleagues that used an ELISA-based approach and found levels ranging from 0 to 15 μg/mL [2,34,35]. Our results are also supported by a recent report from Joral et al., who used the beef thyroglobulin alpha-gal ImmunoCAP assay to study alpha-gal IgG [36]. They found mean levels of 0.5 and 12.3 μg/mL among non-atopic and atopic control subjects recruited from Northern Spain, respectively. One surprising caveat is that only a minor fraction of the controls in their report had detectable levels of alpha-gal IgG (16% of the non-atopics and 31% of the atopics). Nonetheless, the generally similar alpha-gal IgG levels between different cohorts from different countries speak to the utility of ImmunoCAP-based assays in measuring alpha-gal IgG levels. Calculated as a fraction of total IgG, alpha-gal IgG antibodies constituted roughly 0.03% of the total IgG repertoire, significantly lower than some longstanding estimates that alpha-gal IgG constitutes ~1% of the total IgG repertoire [2,34,35]. Differences in alpha-gal IgG values between investigations are likely related to different measurement approaches employed. Prior estimates have used techniques for quantification such as rosette formation and affinity chromatography, with antigens such as rabbit red blood cells and porcine thyroglobulin on the solid phase. However, other factors, including environmental or tick-specific characteristics, and diet or microbiome, cannot be excluded.

The exploration of factors that could promote elevated alpha-gal IgG revealed positive associations for older age, White race, lack of B/AB blood group, alpha-gal IgE sensitization, and a history of tick bites, though age and race lost significance in multiple variable analysis. We found no association between alpha-gal IgG levels and sex or frequency of mammalian meat consumption. To our knowledge, this is one of the first investigations of diet and alpha-gal IgG levels. It has been reported that dietary consumption and food-specific IgG may be correlated for some foods [37]. It is also thought that alpha-gal antibody levels may be impacted by the gut microbiome. Given that meat and dairy consumption could significantly alter the gut microbiome, diet could also have indirect effects on alpha-gal IgG [6,7,38]. Nonetheless, our data suggest any such effects would be modest, as we did not observe differences by diet in this cohort.

It has been previously reported that alpha-gal IgG levels are lower among subjects who express the B blood group antigen (blood types B and AB), likely due to structural similarities between the alpha-gal antigen and the B blood group antigen [39,40,41,42]. This principle may also play a role in reports of lower rates of alpha-gal IgE sensitization among subjects with the B blood group antigen, though no such association was seen in this cohort [28,43,44,45]. The lack of effect of age or sex on alpha-gal IgG is similar to results from Buonomano et al., who studied 200 healthy adults using an ELISA with units expressed in arbitrary units/mL, as well as Bernth-Jensen et al., who employed a time-resolved immunofluorometric assay [42,46].

An association between alpha-gal IgE sensitization and alpha-gal IgG levels has previously been reported. IgG1 predominates, along with smaller increases in alpha-gal IgG2 and IgG3, and no increases in alpha-gal IgG4. Of note, as alpha-gal is a carbohydrate allergen, it tends to elicit a stronger IgG2 response than traditional protein allergens, regardless of IgE sensitization status [26,36,40,43,47]. It is thought that IgG1 and IgG4 antibodies can compete with IgE for antigen binding, thus assuming a protective role against allergic responses [48,49,50]. Despite the unique IgG subtype profile and the high level of IgG antibodies to alpha-gal, we are not aware of reports that any specific IgG subtype provides a protective role against symptomatic MMA [40,47].

The recent report by Chakrapani et al. assessed levels of alpha-gal IgG and IgG subtypes among a population-based control group in Luxembourg, as well as a cohort of forestry workers using an ELISA assay [26]. This group found that alpha-gal IgG levels and alpha-gal IgG subtype IgG1, IgG2, and IgG3 levels were increased in alpha-gal IgE-sensitized forest workers compared to non-sensitized forest workers. They also reported the novel finding, replicated here, that alpha-gal IgG was higher among subjects with a tick bite history, regardless of whether they were alpha-gal IgE positive. These findings imply that bites from ticks, such as *Amblyomma americanum* in the United States or *Ixodes ricinus* in Europe, can enhance the immune response to alpha-gal even in individuals who have no IgE response to alpha-gal [21,51]. Further research is needed to understand whether the lack of IgE in these subjects is a function of tick or host factors. It is possible that some ticks express lower amounts of factors that act as adjuvants to drive Th2-related responses. Conversely, the lack of IgE could be seen in individuals who are not predisposed to be atopic.

While questions about the relevance of anti-Gal antibodies to health and disease remain to be answered, emerging evidence supports the idea that both IgE and IgG antibodies to the alpha-gal antigen may be associated with inflammatory conditions. For example, reports have described links between both antibody isotypes and the early degradation of mammalian-derived prosthetic cardiac valves. Reports have also noted a link with the severity of coronary artery disease [14,17,18,52,53]. Taken together, these findings imply that tick bites could promote specific humoral immune responses to dietary or xeno-antigens that are linked with chronic inflammatory states. Mechanistic studies and animal models will be important to decipher the cellular and mechanistic basis for these observations.

As upwards of 80 percent of subjects with IgE sensitization to alpha-gal are asymptomatic and continue to eat mammalian meat, we sought to investigate the potential of alpha-gal IgG as an adjunct diagnostic marker to alpha-gal IgE for predicting symptomatic MMA [28,32,54]. To investigate this question, we enriched our AGS patient population by measuring alpha-gal IgG levels in sera from 38 patients from the University of Virginia allergy clinic who had alpha-gal IgE sensitization and self-reported MMA. Among those who were alpha-gal IgE sensitized, we found little difference in alpha-gal IgG levels in those who reported MMA or who tolerated mammalian meat. Consistent with this, alpha-gal IgG levels alone could not successfully predict AGS among sensitized subjects, with an area under the ROC curve of 0.53. Joral et al. recently reported that alpha-gal IgG levels could be used as a marker for predicting clinically relevant AGS, as AGS patients in their cohort had significantly higher alpha-gal IgG levels than control populations, including a “risk” population with tick bite history [36]. However, very few (4%) in their risk cohort were alpha-gal IgE positive. We would also highlight that our findings were supported by Chakrapani et al., who showed no differences in alpha-gal IgG levels between asymptomatic and symptomatic subjects who were alpha-gal IgE-positive [26].

In a prior analysis of the vaccine cohort, we conducted receiver operating characteristic (ROC) analysis and found that alpha-gal sIgE levels and sIgE/total IgE ratios had reasonable performance characteristics for distinguishing between asymptomatic alpha-gal sensitized subjects and AGS patients [28]. In the current analysis, we significantly expanded the number of AGS patients from 7 to 45 subjects, and the ROC analysis showed an ideal alpha-gal IgE cutoff of ≥1.4 IU/mL. Notably, this is similar to the cutoff of ≥1.5 IU/mL from the prior analysis, and it aligns with the ideal cutoffs found by other groups to successfully predict symptomatic AGS [55,56]. We would highlight that while this value may be helpful in predicting symptoms on a population level, there is still substantial patient-to-patient variability, and cut-offs should not be used to exclude an AGS diagnosis in an individual with an otherwise positive alpha-gal IgE test who has a convincing clinical history.

The limitations of this study include a lack of IgG subtype analysis, as not all IgG subtypes are measurable on the ImmunoCAP platform. Additionally, retrospective self-reported diet and tick exposure information may not have the granularity or accuracy to answer important questions regarding diet, tick exposure, and alpha-gal IgG levels. As cells were unavailable for forward typing, ABO blood group status was only determined by reverse typing. However, reverse typing by itself has an accuracy estimated at >99% [57]. Also, the vaccine cohort and the AGS patient cohort had significant demographic differences, such as age and sex. Prior research suggests that these demographic differences are likely representative of the risk factors for alpha-gal sensitization and thus AGS. However, future case–control studies that control for these demographic differences could provide valuable insight into the optimal biomarker cutoffs for predicting AGS [28,58,59,60]. Lastly, as the vaccine cohort was mostly white, female, and middle-aged, these findings may have limited generalizability.

In conclusion, we found that—among subjects from a vaccine cohort who were not selected due to history of MMA, allergic disease, or tick exposure –alpha-gal IgG levels were higher among subjects who were alpha-gal IgE-sensitized and subjects who were blood type A or O. Alpha-gal IgG levels constituted less than 0.1% of the total IgG repertoire and, among those who were IgE-sensitized, were not helpful for distinguishing subjects with or without MMA. However, even among those who lacked alpha-gal IgE sensitization, subjects who reported tick bites had higher alpha-gal IgG levels than subjects who did not report tick bites, suggesting a potential link between tick bites and a pro-inflammatory immune response to this dietary antigen.

## Figures and Tables

**Table 1:** Characteristics of the COVID-19 vaccine and AGS patient cohorts.

Characteristics	COVID Cohort (*n* = 267)	AGS Cohort (*n* = 38)	*p*
Age, y (median [interquartile range])	42 (31–55)	62 (52–73)	<0.001
Female sex, *n* (%)	202 (75.7%)	18 (47.4%)	<0.001
Race and Ethnicity			0.24
Asian, *n* (%)	26 (9.7%)	1 (2.6%)	
White, *n* (%)	216 (80.9%)	29 (76.3%)	
Black, *n* (%)	18 (6.7%)	2 (5.3%)	
Hispanic, *n* (%)	6 (2.3%)	0 (0%)	
Other, *n* (%)	1 (0.4%)	1 (2.6%)	
Total IgE, IU/mL (geometric mean [95% CI])	22.5 [18.6–27.2]	106.2 [71.82–157.1]	<0.001
Mammalian Meat Allergy, *n* (%)	8 (3.0%)	38 (100%)	<0.001

**Table 2:** Multiple linear regression model analysis assessing the association between demographic factors and log-transformed alpha-gal IgG levels among subjects with a complete dietary data set (*n* = 263).

Variable	N (%)	Adjusted Linear Regression β (95% CI)	*p*-Value
Age 50+	93 (35%)	0.05 (−0.13 to 0.22)	0.60
Male sex	65 (24%)	−0.01 (−0.19 to 0.17)	0.90
White race	216 (81%)	0.08 (−0.12 to 0.27)	0.46
B or AB blood group	56 (21%)	−0.30 (−0.49 to −0.12)	0.002
Alpha-gal IgE ≥ 0.1 IU/mL	43 (16%)	0.53 (0.32 to 0.75)	<0.001
1–2 Red meat servings/week	129 (48%)	0.004 (−0.19 to 0.20)	0.97
≥3 Red meat servings/week	77 (29%)	0.03 (−0.19 to 0.24)	0.80

**Table 3:** Multiple linear regression model analysis assessing the association of tick/chigger bites and other demographic factors with log-transformed alpha-gal IgG levels among alpha-gal IgE negative subjects (*n* = 166).

Variable	N (%)	Unadjusted Linear Regression β (95% CI)	*p*-Value	Adjusted Linear Regression β (95% CI)	*p*-Value
Age 50+	48 (29%)	0.03 (−0.19 to 0.26)	0.78	0.009 (−0.21 to 0.23)	0.93
Male Sex	40 (24%)	0.17 (−0.07 to 0.40)	0.16	0.19 (−0.04 to 0.42)	0.10
White Race	127 (77%)	0.04 (−0.19 to 0.29)	0.69	0.12 (−0.30 to 0.18)	0.64
B or AB blood group	37 (22%)	−0.42 (−0.65 to −0.18)	<0.001	−0.41 (−0.65 to −0.18)	<0.001
Tick/chigger bite	97 (58%)	0.25 (0.04 to 0.45)	0.02	0.11 (0.04 to 0.46)	0.02

## Data Availability

The data are available upon reasonable request from the authors.
